# Supplementary material for: Huntingtin’s spherical solenoid structure enables polyglutamine tract-dependent modulation of its structure and function
Source: eLife. 2016 Mar 22;5:e11184. doi: 10.7554/eLife.11184 (PMC4846397; doi:10.7554/eLife.11184)
Supplement: Supplementary file 1. — Huntingtin amino acid sequences of crosslinked peptides indicating the relative position of the linked lysines; Position1, Position2, absolute amino acid position of linked lysines. DOI: http://dx.doi.org/10.7554/eLife.11184.016 [file elife-11184-supp1.docx]

**Supplementary file 1. The list of DSS modified peptides of human Q23- (A), Q46- (B) and Q78- (C) huntingtin identified in XL-MS.**

Huntingtin amino acid sequences of crosslinked peptides indicating the relative position of the linked lysines; Position1, Position2, absolute amino acid position of linked lysines.

**A**

| **Cross-linked Peptide ID** | **Position1** | **Position2** |
| --- | --- | --- |
| KDRVNHCLTICENIVAQSVR-KGSEASAASR-a1-b1 | 99 | 1204 |
| MVADECLNKVIK-KGSEASAASR-a9-b1 | 155 | 1204 |
| LQLELYKEIK-KNGAPR-a7-b1 | 174 | 178 |
| TSKRPEESVQETLAAAVPK-VLLKAFIANLK-a3-b4 | 220 | 255 |
| TSKRPEESVQETLAAAVPK-AFIANLKSSSPTIR-a3-b7 | 220 | 262 |
| TSKRPEESVQETLAAAVPK-GKVLLGEEEALEDDSESR-a3-b2 | 220 | 444 |
| DTSLKGSFGVTR-ADKNAIHNHIR-a5-b3 | 337 | 1417 |
| GKVLLGEEEALEDDSESR-QIKNLPEETFSR-a2-b3 | 444 | 1735 |
| QPSDSSVDKFVLRDEATEPGDQENKPCR-IKGDIGQSTDDDSAPLVHCVR-a9-b2 | 648 | 669 |
| IKGDIGQSTDDDSAPLVHCVR-NSSMALQQAHLLKNMSHCR-a2-b13 | 669 | 633 |
| IKGDIGQSTDDDSAPLVHCVR-DEATEPGDQENKPCR-a2-b12 | 669 | 664 |
| IKGDIGQSTDDDSAPLVHCVR-LLSASFLLTGGKNVLVPDR-a2-b12 | 669 | 700 |
| DESSVTCKLACTAVR-QPSDSSVDKFVLR-a8-b9 | 826 | 648 |
| DESSVTCKLACTAVR-ADKNAIHNHIR-a8-b3 | 826 | 1417 |
| DESSVTCKLACTAVR-GTNKADAGK-a8-b4 | 826 | 1559 |
| GAHHYTGLLKLQER-LVSFLEAKAENLHR-a10-b8 | 904 | 888 |
| LFYKCDQGQADPVVAVAR-LVSFLEAKAENLHR-a4-b8 | 943 | 888 |
| LFYKCDQGQADPVVAVAR-GAHHYTGLLKLQER-a4-b10 | 943 | 904 |
| LFYKCDQGQADPVVAVAR-GTNKADAGK-a4-b4 | 943 | 1559 |
| LFYKCDQGQADPVVAVAR-CENLSTPTMLKK-a4-b11 | 943 | 1973 |
| LFYKCDQGQADPVVAVAR-KGFPCEAR-a4-b1 | 943 | 2969 |
| GKEKEPGEQASVPLSPK-KGSEASAASR-a2-b1 | 1188 | 1204 |
| EKEPGEQASVPLSPK-KGSEASAASR-a2-b1 | 1190 | 1204 |
| KGSEASAASR-TNLTSVTKNR-a1-b8 | 1204 | 1412 |
| LHDVLKATHANYK-KELSATK-a6-b1 | 1246 | 92 |
| LHDVLKATHANYK-MVADECLNKVIK-a6-b9 | 1246 | 155 |
| LHDVLKATHANYK-KGSEASAASR-a6-b1 | 1246 | 1204 |
| VTLDLQNSTEKFGGFLR-KGSEASAASR-a11-b1 | 1264 | 1204 |
| TLFGTNLASQFDGLSSNPSKSQGR-TNLTSVTKNR-a20-b8 | 1339 | 1412 |
| VSTQLKTNLTSVTK-KGSEASAASR-a6-b1 | 1404 | 1204 |
| VSTQLKTNLTSVTK-NRADKNAIHNHIR-a6-b5 | 1404 | 1417 |
| VSTQLKTNLTSVTK-GTNKADAGK-a6-b4 | 1404 | 1559 |
| TNLTSVTKNR-GTNKADAGK-a8-b4 | 1412 | 1559 |
| ADKNAIHNHIR-TNLTSVTKNR-a3-b8 | 1417 | 1412 |
| ALKQYTTTTCVQLQK-KGSEASAASR-a3-b1 | 1436 | 1204 |
| KAVTHAIPALQPIVHDLFVLR-GTNKADAGK-a1-b4 | 1535 | 1559 |
| GTNKADAGK-KGFPCEAR-a4-b1 | 1559 | 2969 |
| ADAGKELETQK-ADKNAIHNHIR-a5-b3 | 1564 | 1417 |
| ADAGKELETQK-ENEDKWKR-a5-b5 | 1564 | 1604 |
| ELETQKEVVVSMLLR-GTNKADAGK-a6-b4 | 1570 | 1559 |
| LRDGDSTSTLEEHSEGKQIK-GTNKADAGK-a17-b4 | 1732 | 1559 |
| TNTPKAISEEEEEVDPNTQNPK-TLKDESSVTCK-a5-b3 | 2339 | 818 |
| NKPLKALDTR-EKVSPGR-a2-b2 | 2536 | 2934 |
| NKPLKALDTR-KLSIIR-a5-b1 | 2539 | 2548 |
| NKPLKALDTR-EKVSPGR-a5-b2 | 2539 | 2934 |
| AAAVLGMDKAVAEPVSR-KLSIIR-a9-b1 | 2759 | 2548 |
| LDAESLVKLSVDRVNVHSPHR-EKVSPGR-a8-b2 | 2903 | 2934 |
| LDAESLVKLSVDR-KGFPCEAR-a8-b1 | 2903 | 2969 |
| EKVSPGR-KLSIIR-a2-b1 | 2934 | 2548 |
| KGFPCEAR-NVHKVTTC-a1-b4 | 2969 | 3140 |
| KGFPCEAR-EKVSPGR-a1-b2 | 2969 | 2934 |

**B**

| **Cross-linked Peptide ID** | **Position1** | **Position2** |
| --- | --- | --- |
| LMKAFESLK-KELSATKK-a3-b1 | 9 | 92 |
| KDRVNHCLTICENIVAQSVR-KGSEASAASR-a1-b1 | 99 | 1204 |
| MVADECLNKVIK-ELSATKK-a9-b6 | 155 | 98 |
| LQLELYKEIK-KNGAPR-a7-b1 | 174 | 178 |
| FAELAHLVRPQKCRPYLVNLLPCLTR-LQLELYKEIKK-a12-b7 | 203 | 174 |
| TSKRPEESVQETLAAAVPK-VLLKAFIANLK-a3-b4 | 220 | 255 |
| TSKRPEESVQETLAAAVPK-GKVLLGEEEALEDDSESR-a3-b2 | 220 | 444 |
| TSKRPEESVQETLAAAVPK-AFIANLKSSSPTIR-a3-b7 | 220 | 262 |
| TPPPELLQTLTAVGGIGQLTAAKEESGGR-IKGDIGQSTDDDSAPLVHCVR-a23-b2 | 410 | 669 |
| TPPPELLQTLTAVGGIGQLTAAKEESGGR-FVLRDEATEPGDQENKPCR-a23-b16 | 410 | 664 |
| DEATEPGDQENKPCR-QPSDSSVDKFVLR-a12-b9 | 664 | 648 |
| IKGDIGQSTDDDSAPLVHCVR-FVLRDEATEPGDQENKPCR-a2-b16 | 669 | 664 |
| VSVKALALSCVGAAVALHPESFFSK-IKGDIGQSTDDDSAPLVHCVR-a4-b2 | 714 | 669 |
| LVSFLEAKAENLHR-GTNKADAGK-a8-b4 | 888 | 1559 |
| GAHHYTGLLKLQER-LVSFLEAKAENLHR-a10-b8 | 904 | 888 |
| GAHHYTGLLKLQER-KGFPCEAR-a10-b1 | 904 | 2969 |
| LFYKCDQGQADPVVAVAR-GAHHYTGLLKLQER-a4-b10 | 943 | 904 |
| LFYKCDQGQADPVVAVAR-CENLSTPTMLKK-a4-b11 | 943 | 1973 |
| LFYKCDQGQADPVVAVAR-LVSFLEAKAENLHR-a4-b8 | 943 | 888 |
| SSWASEEEANPAATKQEEVWPALGDR-KGSEASAASR-a15-b1 | 1123 | 1204 |
| SSWASEEEANPAATKQEEVWPALGDR-NRADKNAIHNHIR-a15-b5 | 1123 | 1417 |
| GKEKEPGEQASVPLSPK-KGSEASAASR-a2-b1 | 1188 | 1204 |
| GKEKEPGEQASVPLSPK-KGSEASAASR-a4-b1 | 1190 | 1204 |
| EKEPGEQASVPLSPK-ADKNAIHNHIR-a2-b3 | 1190 | 1417 |
| KGSEASAASR-TNLTSVTKNR-a1-b8 | 1204 | 1412 |
| LHDVLKATHANYK-MVADECLNKVIK-a6-b9 | 1246 | 155 |
| LHDVLKATHANYK-KGSEASAASR-a6-b1 | 1246 | 1204 |
| LHDVLKATHANYK-KELSATK-a6-b1 | 1246 | 92 |
| ATHANYKVTLDLQNSTEK-MVADECLNKVIK-a7-b9 | 1253 | 155 |
| TLFGTNLASQFDGLSSNPSKSQGR-TNLTSVTKNR-a20-b8 | 1339 | 1412 |
| TLFGTNLASQFDGLSSNPSKSQGR-VSTQLKTNLTSVTK-a20-b6 | 1339 | 1404 |
| TLFGTNLASQFDGLSSNPSKSQGR-ADKNAIHNHIR-a20-b3 | 1339 | 1417 |
| NMVQAEQENDTSGWFDVLQKVSTQLK-TNLTSVTKNR-a20-b8 | 1398 | 1412 |
| VSTQLKTNLTSVTK-GTNKADAGK-a6-b4 | 1404 | 1559 |
| VSTQLKTNLTSVTK-KGSEASAASR-a6-b1 | 1404 | 1204 |
| VSTQLKTNLTSVTK-NRADKNAIHNHIR-a6-b5 | 1404 | 1417 |
| TNLTSVTKNR-GTNKADAGK-a8-b4 | 1412 | 1559 |
| ADKNAIHNHIR-TNLTSVTKNR-a3-b8 | 1417 | 1412 |
| NRADKNAIHNHIR-GTNKADAGK-a5-b4 | 1417 | 1559 |
| NRADKNAIHNHIR-KGSEASAASR-a5-b1 | 1417 | 1204 |
| KAVTHAIPALQPIVHDLFVLR-GTNKADAGK-a1-b4 | 1535 | 1559 |
| KAVTHAIPALQPIVHDLFVLR-DTSLKGSFGVTRK-a1-b5 | 1535 | 337 |
| GTNKADAGK-ENEDKWK-a4-b5 | 1559 | 1604 |
| ADAGKELETQK-ENEDKWK-a5-b5 | 1564 | 1604 |
| GTNKADAGKELETQK-ENEDKWKR-a9-b7 | 1564 | 1606 |
| ELETQKEVVVSMLLR-GTNKADAGK-a6-b4 | 1570 | 1559 |
| LIQYHQVLEMFILVLQQCHKENEDK-GTNKADAGK-a20-b4 | 1599 | 1559 |
| HSLSSTKLLSPQMSGEEEDSDLAAK-WWAEVQQTPKR-a7-b10 | 1869 | 1861 |
| TNTPKAISEEEEEVDPNTQNPK-TLKDESSVTCK-a5-b3 | 2339 | 818 |
| TNTPKAISEEEEEVDPNTQNPK-LRDGDSTSTLEEHSEGKQIK-a5-b17 | 2339 | 1732 |
| RTNTPKAISEEEEEVDPNTQNPK-EPGEQASVPLSPKK-a6-b13 | 2339 | 1203 |
| NKPLKALDTR-EKVSPGR-a2-b2 | 2536 | 2934 |
| NKPLKALDTR-KLSIIR-a5-b1 | 2539 | 2548 |
| NKPLKALDTR-EKVSPGR-a5-b2 | 2539 | 2934 |
| KLSIIR-NKPLK-a1-b2 | 2548 | 2536 |
| GIVEQEIQAMVSKR-NKPLK-a13-b2 | 2566 | 2536 |
| AAAVLGMDKAVAEPVSR-KGSEASAASR-a9-b1 | 2759 | 1204 |
| LDAESLVKLSVDR-EKVSPGR-a8-b2 | 2903 | 2934 |
| AMAALGLMLTCMYTGKEK-KLSIIR-a16-b1 | 2932 | 2548 |
| EKVSPGR-KLSIIR-a2-b1 | 2934 | 2548 |

**C**

| **Cross-linked Peptide ID** | **Position1** | **Position2** |
| --- | --- | --- |
| KDRVNHCLTICENIVAQSVR-KGSEASAASR-a1-b1 | 99 | 1204 |
| MVADECLNKVIK-ELSATKK-a9-b6 | 155 | 98 |
| MVADECLNKVIK-KGSEASAASR-a9-b1 | 155 | 1204 |
| LQLELYKEIK-KNGAPR-a7-b1 | 174 | 178 |
| TSKRPEESVQETLAAAVPK-VLLKAFIANLK-a3-b4 | 220 | 255 |
| TSKRPEESVQETLAAAVPK-AFIANLKSSSPTIR-a3-b7 | 220 | 262 |
| TSKRPEESVQETLAAAVPK-GKVLLGEEEALEDDSESR-a3-b2 | 220 | 444 |
| IKGDIGQSTDDDSAPLVHCVR-DEATEPGDQENKPCR-a2-b12 | 669 | 664 |
| DESSVTCKLACTAVR-QPSDSSVDKFVLR-a8-b9 | 826 | 648 |
| DESSVTCKLACTAVR-ADKNAIHNHIR-a8-b3 | 826 | 1417 |
| DESSVTCKLACTAVR-GTNKADAGK-a8-b4 | 826 | 1559 |
| LVSFLEAKAENLHR-GTNKADAGK-a8-b4 | 888 | 1559 |
| LFYKCDQGQADPVVAVAR-LVSFLEAKAENLHR-a4-b8 | 943 | 888 |
| LFYKCDQGQADPVVAVAR-GAHHYTGLLKLQER-a4-b10 | 943 | 904 |
| LFYKCDQGQADPVVAVAR-GTNKADAGK-a4-b4 | 943 | 1559 |
| LFYKCDQGQADPVVAVAR-CENLSTPTMLKK-a4-b11 | 943 | 1973 |
| LFYKCDQGQADPVVAVAR-KGFPCEAR-a4-b1 | 943 | 2969 |
| SSWASEEEANPAATKQEEVWPALGDR-TNLTSVTKNR-a15-b8 | 1123 | 1412 |
| EKEPGEQASVPLSPK-KGSEASAASR-a2-b1 | 1190 | 1204 |
| EPGEQASVPLSPKKGSEASAASR-KGSEASAASR-a13-b1 | 1203 | 1204 |
| KGSEASAASR-TNLTSVTKNR-a1-b8 | 1204 | 1412 |
| LHDVLKATHANYK-MVADECLNKVIK-a6-b9 | 1246 | 155 |
| ATHANYKVTLDLQNSTEK-MVADECLNKVIK-a7-b9 | 1253 | 155 |
| VTLDLQNSTEKFGGFLR-KGSEASAASR-a11-b1 | 1264 | 1204 |
| TLFGTNLASQFDGLSSNPSKSQGR-TNLTSVTKNR-a20-b8 | 1339 | 1412 |
| VSTQLKTNLTSVTK-KGSEASAASR-a6-b1 | 1404 | 1204 |
| VSTQLKTNLTSVTK-ADKNAIHNHIR-a6-b3 | 1404 | 1417 |
| VSTQLKTNLTSVTK-GTNKADAGK-a6-b4 | 1404 | 1559 |
| TNLTSVTKNR-GTNKADAGK-a8-b4 | 1412 | 1559 |
| TNLTSVTKNR-KLSIIR-a8-b1 | 1412 | 2548 |
| ADKNAIHNHIR-TNLTSVTKNR-a3-b8 | 1417 | 1412 |
| ALKQYTTTTCVQLQK-KGSEASAASR-a3-b1 | 1436 | 1204 |
| KAVTHAIPALQPIVHDLFVLR-GTNKADAGK-a1-b4 | 1535 | 1559 |
| GTNKADAGK-KGFPCEAR-a4-b1 | 1559 | 2969 |
| ELETQKEVVVSMLLR-GTNKADAGK-a6-b4 | 1570 | 1559 |
| TNTPKAISEEEEEVDPNTQNPK-TLKDESSVTCK-a5-b3 | 2339 | 818 |
| TNTPKAISEEEEEVDPNTQNPK-KGSEASAASR-a5-b1 | 2339 | 1204 |
| TNTPKAISEEEEEVDPNTQNPK-KLSIIR-a5-b1 | 2339 | 2548 |
| NKPLKALDTR-KLSIIR-a5-b1 | 2539 | 2548 |
| NKPLKALDTR-EKVSPGR-a5-b2 | 2539 | 2934 |
| AAAVLGMDKAVAEPVSR-KLSIIR-a9-b1 | 2759 | 2548 |
| LDAESLVKLSVDR-EKVSPGR-a8-b2 | 2903 | 2934 |
| LDAESLVKLSVDR-KGFPCEAR-a8-b1 | 2903 | 2969 |
| EKVSPGR-KLSIIR-a2-b1 | 2934 | 2548 |
| KGFPCEAR-EKVSPGR-a1-b2 | 2969 | 2934 |
| KGFPCEAR-NVHKVTTC-a1-b4 | 2969 | 3140 |
